# 

**Published:** 1915-01-23

**Authors:** 


### Hospitals and Smoke.

Even in days of war we may recall that the Air Pollution Advisory Board, which was appointed by the Manchester City Council to make an exhaustive investigation into the smoke nuisance in Manchester similar to that carried out in Pittsburg, U.S.A., has drafted a number of schedules of questions to be addressed to institutions, individuals, and firms likely to yield useful information. One schedule of questions has been specially prepared for submission to those in charge of hospitals. Amongst the questions which are asked are the following :—State or estimate how much greater your labour and expenses for cleaning are in Manchester than they would be in a city free from a smoky and acid atmosphere. If the city were free from a smoky and acid atmosphere, what part of your present working staff could be dispensed with, and at what saving per annum? What, in your opinion, is the extra yearly cost of your linen bill due to the smoky and acid atmosphere of Manchester? What does the dust in the wards consist of? Does the smoky and acid condition of the atmosphere add to the difficulty of keeping your operating-rooms as clean as they should be? Do you use any air-purifying devices to counteract the effects of the dirty atmosphere in Manchester, and, if so, what is the system used, the original cost, and the annual expenditure for maintenance? What is your estimated cost for extra artificial light per month or per year due to dark or foggy days? Is the yearly cost of washing and laundering bed linen, towels, nurses' costumes, etc., increased by the smoky and acid atmosphere of Manchester, and, if so, by how much? What is the extra yearly cost of cleaning walls, renewing inside decorations, and the like, due to the smoky and acid-laden atmosphere? Does the smoky and acid condition of our Manchester atmosphere cause you to keep the windows closed? Can you estimate the total yearly cost to your institution of the damage directly resulting from the smoky atmosphere of Manchester? If you are familiar with hospital management in other cities, state as completely as possible what losses and inconveniences in Manchester are attributable to the smoky and acid atmosphere. What is your opinion as to the state of the Manchester atmosphere to-day as compared with ten or twenty years ago? Do you favour the adoption of reasonable measures to secure the abatement of the smoke evil? These are all questions which have occurred to the board when considering the possible effect of the smoke nuisance on city hospitals.

### Medical Appointments.

DR. D. R. C. SHEPHERD has been re-elected resident medical officer of the Middlesex Hospital.

MR. ARTHUR CYRIL HUDSON, M.A., M.D., B.C. Cantab., F.R.C.S. Eng., has been appointed ophthalmic surgeon in charge of out-patients to St. Thomas's Hospital.

MR. JOHN BRUNTON, M.D. Glasg., D.P.H. Camb., M.R.C.S., L.R.C.P., has been appointed to a clinical assistantship in the out-patient department at the West End Hospital.

DR. E. H. MORRIS, junior medical officer at Camberwell Infirmary, who is at present abroad attending to the wounded, has been reappointed for twelve months by the Camberwell Board of Guardians. She is receiving half-pay in accordance with the determination of the board. During her absence Dr. Eliza Davidson has been appointed to fill her position at a salary of £250 a year, with the usual indoor allowances.

### Personalia.

### Dr. Isaac Burney Yeo's Bequests.

DR. ISAAC BURNEY YEO, M.D., F.R.C.P., of 8 Cadogan Place, S.W., Emeritus Professor of Medicine at King's College, London, and consulting physician to King's College Hospital, who died on November 20 and left estate valued at £95,413 gross, has bequeathed £3,000 to the Royal Medical Benevolent Fund to found an annuity fund to be known as "The Burney Yeo Bequest"; £3,000 to the Royal Medical Benevolent College, Epsom, for a similar purpose; £5,000 to King's College (London) Hospital Medical School, for a "Burney Yeo" fund to be used for furthering the success of the school. He left the ultimate residue of his estate as to one moiety to the Royal Medical Benevolent Fund and one moiety to the Royal Medical Benevolent College at Epsom.

### MANAGER'S NOTICES.

**All letters on business matters must be addressed to The Manager, "The Hospital," 28 and 29 Southampton Street, London, W.C.**

**Subscriptions**, which may commence at any time, are payable in advance. Cheques and Post-Office Orders should be crossed London County and Westminster Bank, Covent Garden Branch, and made payable to the Manager of THE HOSPITAL.

RATES OF SUBSCRIPTION (payable in advance).

UNITED KINGDOM.

| | |
|---|---|
| Three Months (including Postage) ... ... ... ... ... | 2s. 0d. |
| Six Months do. ... ... ... ... ... | 4s. 0d. |
| Twelve Months do. ... ... ... ... ... | 6s. 6d. |

FOREIGN AND COLONIAL.

| | |
|---|---|
| Three Months (including Postage) ... ... ... ... ... | 2s. 6d. |
| Six Months do. ... ... ... ... ... | 5s. 0d. |
| Twelve Months do. ... ... ... ... ... | 8s. 8d. |

**Remittances:**

**It is especially requested that remittances be made by Cheque or Postal Order, and in halfpenny stamps only when the amount is under sixpence.**

**Advertisements:**

To ensure insertion, all advertisements for THE HOSPITAL must reach the Manager not later than Wednesday at noon in each case. For scale of charges see pages iii and 3.

**Special Rates** will be quoted to those institutions that agree to send all their public notices to THE HOSPITAL, so as to make it a file of such announcements for ready reference.

RATES OF SUBSCRIPTION: Twelve months, 6/6; Six months, 4/-; Three months, 2/-, post free to any address in the United Kingdom. Abroad, Twelve months, 8/8; Six months, 5/-; Three months, 2/6, post free.—Address The Subscription Dept., "THE HOSPITAL," The Hospital Building, 28/29 Southampton Street, Strand, London, W.C.

